# The Clinical Significance of Ependymal Enhancement at Presentation in Patients with Malignant Glioma

**DOI:** 10.5041/RMMJ.10224

**Published:** 2015-10-26

**Authors:** Orit Kaidar-Person, Firas Darawshe, Tzahala Tzuk-Shina, Ayelet Eran

**Affiliations:** 1 Division of Oncology & Neuro-Oncology Unit, Rambam Health Care Campus, Haifa, Israel; 2 Department of Radiology, Rambam Health Care Campus, Haifa, Israel

**Keywords:** Central nervous system, ependymal enhancement, glioblastoma multiforme, malignant glioma, MRI, radiotherapy

## Abstract

**Introduction:**

The current study evaluated the rate of ependymal enhancement and whether its presence influences survival of patients with malignant glioma (GBM).

**Methods:**

A retrospective review of all patients who were treated in our institution from 2005 to 2011 was conducted. Data extracted from the medical records included age, date of diagnosis, co-morbidities, treatment regimen, and time of death. Magnetic resonance images (MRI) were evaluated for the presence of ependymal enhancement and its extent, and the correlation to survival was investigated.

**Results:**

Between 2005 and 2011, 230 patients were treated for GBM. Eighty-nine patients were excluded from the study due to insufficient data, leaving 141 patients for analysis. Median age at diagnosis was 60 years. Sixty-seven (40.6%) patients had evidence of ependymal enhancement on MRI (group A), and 70 (42.4%) patients did not have evidence of enhancement. The assessment of ependymal enhancement was inconclusive due to mass effect and ventricular compression that precluded accurate assessment for 28 (17%) patients (group C). Median survival was 14 months for group A (range, 12–16 months), 15.9 months for group B (range, 14.28–17.65 months), and 11.7 months for group C (range, 6.47–16.92 months) (*P*>0.05). A multivariate analysis to predict survival indicated that male gender (*P*=0.039), hypertension (*P*=0.012), and biopsy only compared to complete gross tumor resection (P=0.001) were significant for poor survival.

**Conclusions:**

Pretreatment ependymal enhancement on MRI was not found to be associated with poorer survival. These results might be due to better treatments options compared to prior reports.

## INTRODUCTION

Glioblastoma multiforme (GBM) is the most common primary central nervous system malignancy in adults. A paradigm shift in the treatment of GBM was a result of the European Organization for Research and Treatment of Cancer (EORTC) and the National Cancer Institute of Canada trial, which was a randomized prospective trial comparing surgery followed by either radiation therapy alone, or radiation therapy plus the addition of concurrent daily temozolomide and six months of adjuvant temozolomide (RT+TMZ).^1^,^2^ However, 14.6 months as median survival of the study group has a range of 13.2–16.8 months, a difference that is greater than that of the two study groups, implying that there are other factors affecting survival and that there are subgroups of patients with different disease aggressiveness. Molecular and imaging factors are constantly investigated in order to identify subgroups of patients with different aggressiveness of disease and potentially offer different treatment paradigms.

Magnetic resonance imaging (MRI) is commonly utilized as part of the diagnostic workup for the clinical diagnosis and treatment planning of GBM. The role of MRI in treatment planning and evaluating the aggressiveness of the disease is constantly evolving.^3^ Prior studies suggest that GBM bordering the ventricular system are associated with a more aggressive and multifocal phenotype and may be associated with increased recurrence rate and morbidity,^4^–^7^ while other studies did not show such an association.^8^

The current study was designed to evaluate the rate of ependymal enhancement and to determine whether its presence influences survival of patients treated with surgery (when possible) and RT+TMZ for GBM.

## METHODS

After institutional review board approval, a retrospective review of all patients who were treated in our institution from 2005 to 2011 was conducted. Only patients whose pretreatment MRI was available were included in our study. Data extracted from the medical records included age, date of diagnosis, co-morbidities, Eastern Cooperative Oncology Group (ECOG) performance status score at time of first visit prior to initiating treatment by RT+TMZ, extent of resection, symptoms, steroid use, treatment regimen, time of death, and more. All MRI studies were reviewed by a neuroradiologist with more than 10 years of experience and blinded to patient outcomes (author A.E.). Images were evaluated for the presence of ependymal enhancement and its extent, distance from ventricular wall, cortical enhancement, and leptomeningeal enhancement. Any questionable case was discussed with another investigator (author T.T.S., neuro-oncologist) and solved by consensus.

### Statistical Analysis

Time-to-tumor progression (TTP) was defined as the time from the first day of treatment to the first recorded evidence of progression or change of chemotherapy line. Alive patients without progression were censored at last follow-up. Overall survival (OS) was defined as the time from the first day of treatment to death (all causes). Survivors were censored at the last follow-up.

Bivariable Cox regression was used for the calculation of the hazard ratios (HR) with 95% confidence intervals (CI) and *P* values for factors of OS and TTP. Multivariable Cox regression analysis was performed to assess the relation between patients’ characteristics and outcomes. All variables with a *P* value of ≥0.2 in univariate analysis were selected as candidates for the multivariable analysis. A Kaplan–Maier curve was used to illustrate survival. Two-tailed *P* values of 0.05 or less were considered as statistically significant. Statistical analysis was performed using SPSS (Statistics Products Solutions Services) 21.0 software for Windows, made by the biostatistician service at our institution.

## RESULTS

**Figure 1:**
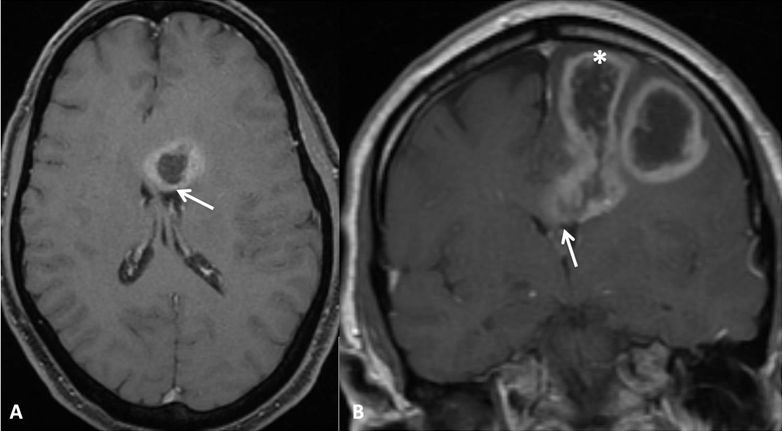
Evidence of Ependymal Enhancement on MRI (Group A) Axial **(A)** and coronal **(B)** gadolinium-enhanced T1-weighted images showing an enhancing tumor extending along the left frontal horn (arrow). Note cortical enhancement (asterisk, B), also present in this case.

**Figure 2:**
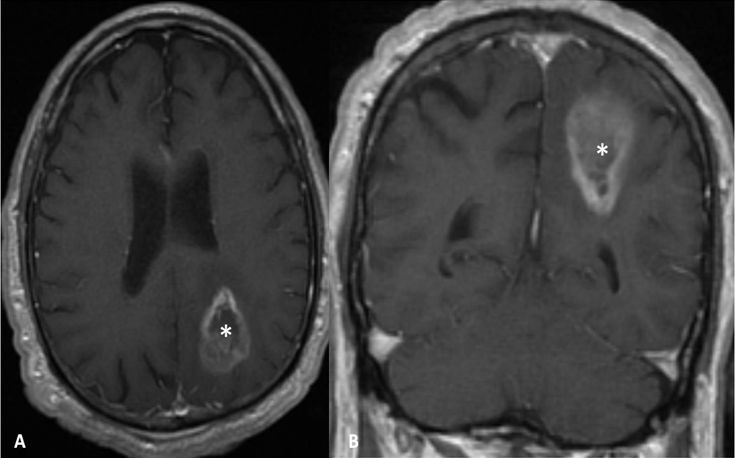
No Evidence of Ependymal Enhancement on MRI (Group B) **Axial (A)** and coronal **(B)** gadolinium-enhanced T1-weighted images showing a left parietal ring enhancing lesion not contacting the ventricular wall.

**Figure 3:**
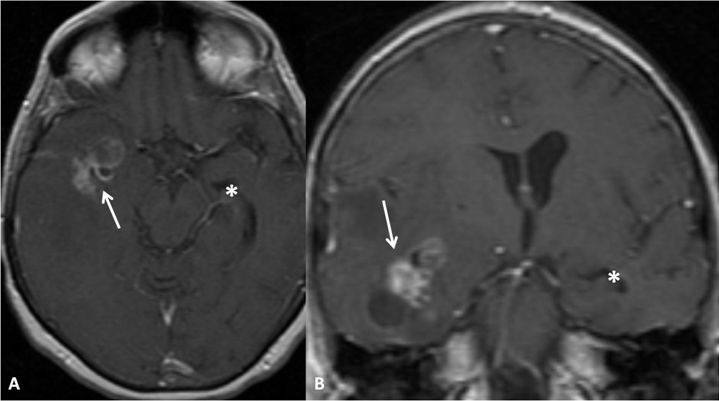
Inconclusive for Ependymal Enhancement on MRI (Group C) Axial **(A)** and coronal **(B)** gadolinium-enhanced T1-weighted images showing a right temporal heterogeneously enhancing mass (arrow) compressing the right temporal horn, thereby making it impossible to appreciate ependymal enhancement along it. Note the left temporal horn (asterisk) at the same level.

Between 2005 and 2011, 230 patients were treated for GBM in the Neuro-Oncology Unit of our hospital. Eighty-nine patients were excluded from the study due to insufficient data, leaving 141 patients for analysis. Median age at diagnosis was 60 years. At the time of data collection, 14% of the patients were still alive. Sixty-seven (40.6%) patients had evidence of ependymal enhancement on MRI (group A) (Figure 1) at a range of 0.2–10 cm of longest diameter of enhancement. Seventy (42.4%) patients did not have evidence of enhancement (group B) (Figure 2). The minimal distance from ventricular wall in this group was 0.2 cm (range, 0.2–3.4 cm). In 28 (17%) patients, assessment of ependymal enhancement was inconclusive due to mass effect and ventricular compression that precluded accurate assessment. This was usually encountered near the temporal horn and defined group C (Figure 3). Median survival of all groups was 15.3 months (range, 13.6–16.95 months).

Median survival according to ependymal enhancement was 14 months for group A (range, 12–16 months), 15.9 months for group B (range, 14.28–17.65 months), and 11.7 months for group C (range, 6.47–16.92 months) (*P*>0.05).

**Figure 4:**
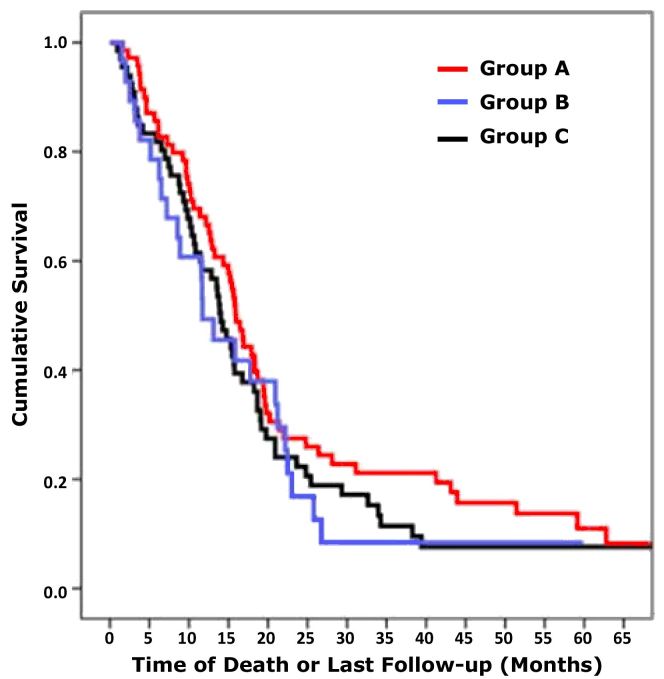
Kaplan–Maier Survival Plots for Different Groups **Group A:** Evidence of ependymal enhancement on MRI. **Group B:** No evidence of ependymal enhancement on MRI. **Group C:** Ependymal enhancement on MRI inconclusive.

Figure 4 summarizes the Kaplan–Maier plot for the different groups. When uniting group A and group C versus group B (enhancement and inconclusive for enhancement versus no enhancement), there were still no differences in survival (*P*>0.05). A year after diagnosis, 59.7% of patients in group A were alive compared to 67.1% in group B, and 50% in group C. These differences were also found to be insignificant (*P*=0.28).

Cox regression test univariate analysis of clinical factors (other than ependymal enhancement) to predict survival is presented in Table 1. A multivariate analysis to predict survival indicated that male gender (*P*=0.039, HR=2.6, 95% CI 1.048–6.5), hypertension (*P*=0.012, HR=3.2, 95% CI 1.13–9.4), and biopsy only compared to complete gross tumor resection (*P*=0.001, HR=1.9, 95% CI 1.28–2.88) were significant for poor survival.

**Table 1:** Univariate Analysis of Clinical Factors to Predict Survival.

Parameter (*n*)		Median Survival	*P* Value
**Gender**	Female (*n*=63)	15.4 months	0.88
	Male (*n*=102)	15.2 months	

**Cortical Involvement**	Yes (*n*=121)	15.4 months	0.044
	No (*n*= 44)	13.2 months	

**Extent of Resection**	Total (*n*=105)	16.8 months	<0.0001 between total and biopsy only group
	Subtotal (*n*=14)	16.7 months	
	Biopsy only (*n*=44)	9.2 months	

**Radiotherapy With and Without TMZ**	RT+TMZ (*n*=152)	15.3 months	0.79
	RT only (*n*=13)	14.7 months	

**ECOG Performance Status**	PS-0 (*n*=56)	19.6 months	<0.005 for all groups
	PS-1 (*n*=47)	18.2 months	
	PS-2 (*n*=36)	11.4 months	
	PS-3 (*n*=18)	9.6 months	
	PS-4 (*n*=8)	5.8 months	

**Hypertension**	Yes (*n*=71)	11.7 months	0.05
	No (*n*=94)	16.7 months	

**Diabetes Mellitus**	Yes (*n*=25)	10.16 months	0.016
	No (*n*=140)	15.76 months	

**Seizures at Time of Diagnosis**	Yes (*n*=46)	16.8 months	0.16
	No (*n*=119)	14.26 months	

**Vomiting at Time of Diagnosis**	Yes (*n*=18)	15.7 months	0.21
	No (*n*=147)	15.2 months	

**Visual Disturbances at Time of Diagnosis**	Yes (*n*=14)	23.6 months	0.015
	No (*n*=151)	14.2 months	

**Headaches at Time of Diagnosis**	Yes (*n*=84)	15.9 months	0.02
	No (*n*=81)	12.9 months	

**Motor Deficit at Time of Diagnosis**	Yes (*n*=60)	15.4 months	0.17
	No (*n*=105)	15.2 months	

**Sensory Deficit at Time of Diagnosis**	Yes (*n*=9)	15.4 months	0.84
	No (*n*=156)	15.2 months	

**Cognitive Impairment/Confusion at Time of Diagnosis**	Yes (*n*=57)	12.5 months	0.04
	No (*n*=108)	15.8 months	

**Steroid-dependent**	Yes (*n*=157)	15.2 months	0.45
	No (*n*=8)	16.9 months	

RT, radiotherapy; RT+TMZ, adjuvant temozolomide and radiotherapy according to Stupp et al.^1^

## DISCUSSION

Our study evaluated whether GBM with ependymal wall enhancement is associated with shorter survival. As opposed to prior reports,^4^–^7^ our study failed to show a significant association. The majority of patients in our study were at good performance status (the performance status of 60% of the patients was defined as ECOG 0–1), which is a well-established prognostic factor and is incorporated in the recursive partitioning analysis (RPA). In prior studies that reported correlation between ependymal enhancement and worse outcome,^5^–^7^ there was a low or non-reported rate of RT+TMZ treatment. However, almost all the patients in our cohort were treated with RT+TMZ, and four patients received a second line with bevacizumab (data not shown). This difference might explain our distinct results. Young et al.^4^ studied a group of patients that all received RT+TMZ and found that periventricular enhancement was associated with worse prognosis; however, their group consisted of patients who had partial resection or biopsy and, therefore, represent a distinct group of patients. Surprisingly, no differences in survival were found between patients who were treated with RT+TMZ and RT only; however, the RT only group was significantly smaller.

Our initial plan was to divide the patients into two groups, with or without evidence of ependymal enhancement. However, some of the cases were inconclusive for involvement, and we had to create a third group, although this had no significant influence on the results.

Cox regression univariate analysis of clinical factors indicated interesting results. Some of the parameters associated with better survival were symptoms at presentation. Visual disturbances and severe headaches at presentation were associated with better prognosis. This might be due to diagnosis at an earlier stage rather than extensive disease. This might also explain the correlation of cortical enhancement and better survival. This parameter was not expected to be associated with better survival, since it might enhance meningeal involvement and in prior studies^5^ was not found to be associated with a better phenotype. A possible explanation of the association between cortical involvement and better survival is that it might be associated with earlier symptoms, such as seizures or functional deficiency, leading to diagnosis. Diabetes mellitus and hypertension were both associated with poorer prognosis.

Our study has some limitations. It is a retrospective study with a limited number of patients. During the study period there were 230 GBM patients, but only 141 were available for analysis, and, of these, in 28 cases the ependymal enhancement was inconclusive for involvement. Thus, more studies are needed to confirm these results.

In summary, according to our study, pretreatment ependymal enhancement on MRI in patients who were diagnosed with GBM was not found to be associated with poorer survival. These results might be due to better treatments options compared to prior reports.

## Abbreviations

CI: confidence intervals
GBM: glioblastoma multiforme
HR: hazard ratios
MRI: magnetic resonance imaging
OS: overall survival
RT+TMZ: radiation therapy + daily temozolomide and six months of adjuvant temozolomide
TTP: time-to-tumor progression.
